# Ectodermal Dysplasia Associated with Sickle Cell Disease

**DOI:** 10.1155/2014/314391

**Published:** 2014-09-29

**Authors:** Luiz Evaristo Ricci Volpato, Maria Carmen Palma Faria Volpato, Artur Aburad de Carvalhosa, Vinicius Canavarros Palma, Álvaro Henrique Borges

**Affiliations:** Department of Post-Graduation, Master Program in Integrated Dentistry Science, University of Cuiabá, Avenida Manoel José de Arruda No. 3.100, 78050-000 Cuiabá, MT, Brazil

## Abstract

Ectodermal dysplasia and sickle cell anaemia are inherited disorders that affect, respectively, the tissues derived from the embryonic ectoderm and the production of erythrocytes by the bone marrow. The simultaneous occurrence of both disorders is extremely rare. This is a case of both ectodermal dysplasia and sickle cell anaemia reported in a 6-year-old. The patient had been diagnosed with sickle cell anaemia for only six months when he sought treatment presenting with the following: hypotrichosis, dry skin, periocular hyperpigmentation, protruding lips, hypodontia, and morphologically altered teeth. The clinical features combined with his medical history led to the diagnosis of ectodermal dysplasia. Dentists should be prepared to recognise patterns that escape normality to aid in the diagnosis of systemic changes, even in patients with other previous diagnoses.

## 1. Introduction

Ectodermal dysplasia comprises a large and heterogeneous group of inherited disorders defined by primary defects in the development of two or more tissues derived from the embryonic ectoderm. Ectodermal dysplasia is classified according to the combination and severity of the presented features [1]. The various forms of this disorder can be inherited in any of several genetic patterns: autosomal dominant, autosomal recessive, and X-linked [2].

It is a rare condition affecting 1 : 10,000 to 1 : 100,000 live births at a rate of five men to one woman and is usually manifested in men but transmitted by women [3, 4]. Its clinical manifestations may vary among individuals, but the condition is usually characterised by hypotrichosis, hypohidrosis, and hypodontia [4]. Other signs of the disorder include hyperpigmentation of the periocular skin and midface hypoplasia resulting in protruding lips and brittle and dystrophic nails [2].

Sickle cell anaemia is an inherited disease and is the most prevalent disorder among the sickle cell syndromes. It is a clinically severe condition affecting millions of individuals around the world and often producing fatal symptoms [5]. The disease results from an inadequate production of erythrocytes by the bone marrow, which limits the replacement of circulating blood cells every 120 days. Sickle cell anaemia results in fragile erythrocytes that are unable to survive for more than 20 days. Thus, the bone marrow is unable to keep up with the continuous cell production demand. This ultimately results in a low oxygen-carrying capacity, accumulation of waste, and risk of hypoxia throughout the body. This disease has no known cure. Affected patients receive symptomatic treatment for pain relief [6].

A search of the Medline database using the terms “sickle cell anemia” and “ectodermal dysplasia” was performed and did not find any reference to the simultaneous occurrence of these diseases in one individual.

## 2. Case Description

**Figure 1:**
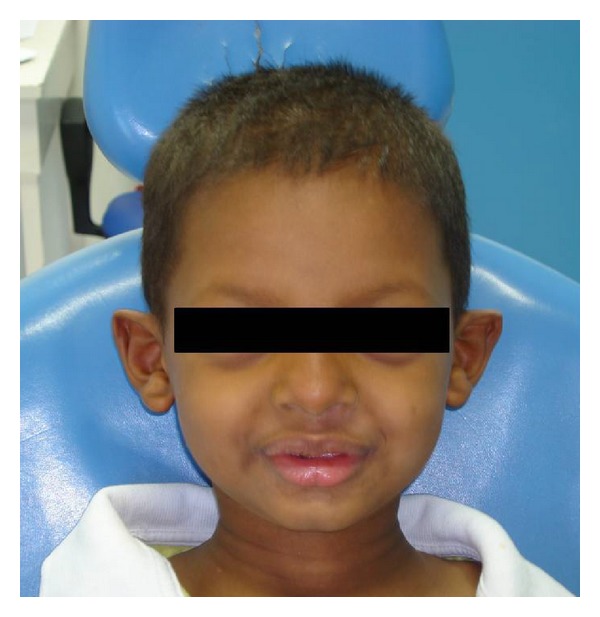
The patient exhibited hypotrichosis, dry skin, periocular hyperpigmentation, and protruding lips.

**Figure 2:**
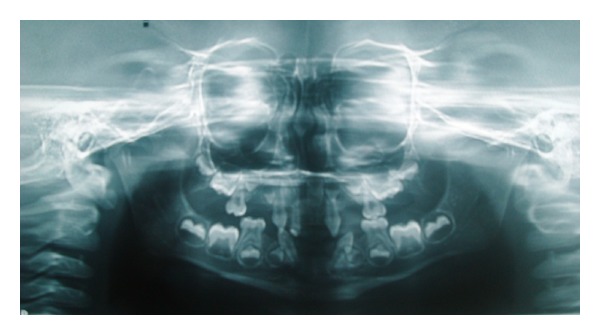
Panoramic radiography revealed the congenital absence of all deciduous first molars, lateral incisors, and lower central incisors; absence of germs in all first premolars, lateral incisors, and lower central incisors; and morphological alterations in deciduous canines and second molars.

A 6-year-old melanoderm boy with sickle cell anaemia was accompanied by his mother and sought treatment for multiple missing teeth. The extraoral examination revealed features of ectodermal dysplasia: hypotrichosis, dry skin, hypodontia, periocular hyperpigmentation, and protruding lips (Figure 1). At the time of the intraoral examination, the patient presented with the congenital absence of deciduous first molars and upper lateral incisors and the absence of first molars and central and lateral incisors in the lower arch. The canines and second molars also displayed morphological alterations. Panoramic radiography showed that the germs of all first premolars, lateral incisors, and lower central incisors were missing (Figure 2).

The patient's mother reported that the patient had been treated for chronic anaemia since the age of 6 months. She also reported frequent episodes of fever and respiratory infections. Only six months before our examination, the patient was referred for specialised treatment due to his diagnosis of sickle cell anaemia. The mother presented a recent blood test confirming the presence of haemoglobin variant HB-S and positive proof of sickling.

## 3. Discussion

Although uncommon, the manifestation of ectodermal defects in association with other anomalies is not rare [7, 8]. These combinations are called ectodermal dysplasia syndromes [8].

In the presented case, the mother had only received her son's diagnosis of sickle cell disease six months before and had not yet been informed of her son's ectodermal dysplasia, despite his clinical features.

The diagnosis of ectodermal dysplasia can be achieved by skin biopsy (Juhlin's test), which detects abnormal distributions of the sweat glands and hair follicles, which are characteristic of this syndrome [9]. According to Kupietzky and Houpt [3], reports of episodes of fever in the first two years of life, abnormal morphology and delayed tooth eruption, peeling skin, eczema, and asthma or frequent respiratory infections may indicate the possibility of a positive diagnosis. The diagnosis is commonly made by clinical observation in combination with the family's medical history [8].

Apparently, there is no relationship between the aetiologies of both disorders. The simultaneous occurrence may have happened by chance; however, the patient was referred to a geneticist for further investigation. The patient is currently being treated for symptoms of sickle cell anaemia and was referred for oral rehabilitation.

In fact, patients with ectodermal dysplasia undergo severe social problems and suffer from poor psychological and physiological development as a result of unacceptable aesthetics and abnormal function of orofacial structures. Oral rehabilitation thus becomes mandatory [10] and involves interdisciplinary approach [11, 12], comprising of dermatologist, psychiatrist, stomatologist, orthodontist, prosthodontist, and pedodontist [10].

Treatment should be aimed at maintaining the dentition present and alveolar ridges as these structures may have to support the denture prosthesis for a life time [12]. Since oligodontia or anodontia leads to atrophy of the alveolar ridges, reduced vertical dimension, prominent chin, and Class III intermaxillary relationship, early prosthetic treatment should be performed as soon as possible [13]. The prosthetic treatment should be carried out on individual basis, aimed always toward providing good occlusal stability. It also aids in phonation and mastication. These factors instil greater self confidence in the child, help him gain acceptance [12], and have positive effects on social development, self-image, and food choice [11], improving his quality of life [13].

Oral rehabilitation may include orthodontic treatment [11], composite build-up [11, 12], crowns [12], fixed prosthesis [11, 12], overdentures [12], removable prosthesis [12], or implants [11, 12] depending on the dentition present [12] and the patient's age and osseous maturity. Implant-supported denture is suggested as the ideal reconstruction modality for adolescents over 12 years [13] because of its bone preserving ability and enhanced retention of the prosthesis [11]. Early implant placement in a growing child may cause cosmetic problems because implants act similar to ankylosed teeth. Along with the craniofacial growth, implant overstructures may not be in occlusion with opposite teeth and even the adjacent teeth may tilt into the space [13].

However, patient's rehabilitation is often difficult [10, 13] because of the typical oral deficiencies [13] and the young age when they are evaluated for treatment [10, 13], demanding knowledge of behavioural management of the paediatric patient [12]. Therefore, when treating a child with ectodermal dysplasia, it is important to motivate both the child and his parents prior to the treatment and to work with them to ensure their compliance [12]. In the present case it could be even more complicated due to the simultaneous occurrence of sickle cell anemia and its complications. Success will depend on regular recall appointments and meticulous maintenance of oral and prosthetic hygiene [13].

## 4. Conclusion

Dentists should be prepared to recognise patterns that escape normality to aid in the diagnosis of systemic changes, even in patients with other previous diagnoses. In the presented case, in addition to the clinically visible alterations of ectodermal dysplasia, the patient also had sickle cell anaemia, a condition that could have been overlooked if professionals took into account only the clinical aspect of the patient.
